# Associations with lip cant and facial midline correction following bimaxillary surgery in class III asymmetry: A CBCT-based analysis

**DOI:** 10.1016/j.bj.2025.100877

**Published:** 2025-06-12

**Authors:** Chih-Ling Lin, Yun-Fang Chen, Ying-An Chen, Chuan-Fong Yao, Tong Xi, Yu-Fang Liao, Yu-Ray Chen

**Affiliations:** a Department of Dentistry, New Taipei Municipal Tu Cheng Hospital, New Taipei, Taiwan; b Department of Craniofacial Orthodontics, Chang Gung Memorial Hospital, Taipei, Taiwan; c Craniofacial Research Center, Chang Gung Memorial Hospital at Linkou, Taoyuan, Taiwan; d College of Medicine, Chang Gung University, Taoyuan, Taiwan; e Department of Plastic and Reconstructive Surgery, Chang Gung Memorial Hospital at Linkou, Taoyuan, Taiwan; f Department of Oral and Maxillofacial Surgery, Radboud University Nijmegen Medical Centre, the Netherlands; g Department of Craniofacial Orthodontics, Chang Gung Memorial Hospital, Taoyuan, Taiwan

**Keywords:** Lip cant, Orthognathic surgery, Class III malocclusion, Facial asymmetry, Association factors

## Abstract

**Background:**

This study evaluated the outcomes of bimaxillary surgery for class III asymmetry and lip cant, and identified factors associated with lip cant and facial midline correction.

**Materials and methods:**

Fifty adult patients (22 females, 28 males; mean age: 24.8 ± 5.1 years) with class III asymmetry and lip cant who underwent bimaxillary surgery were prospectively and consecutively analyzed. Cone-beam computed tomography scans obtained preoperatively and at postoperative follow-up were superimposed to assess surgical jaw movements in six degrees of freedom and their effects on lip cant and facial midline symmetry.

**Results:**

Significant reductions were observed in lip cant (1.6 ± 1.6 mm), lower lip deviation (2.4 ± 1.7 mm), chin deviation (5.8 ± 4.2 mm), and facial midline deviation (9.7 ± 7.2 mm). Multiple linear regression analysis identified mandibular roll correction (β = 0.456, *p* < 0.01) and pre-treatment lip cant severity (β = 0.394, *p* < 0.01) as significant factors of lip cant reduction. Additionally, chin shift (β = 0.495, *p* < 0.01) and mandibular shift (β = 0.461, *p* < 0.01) were significant factors of facial midline correction.

**Conclusion:**

Bimaxillary surgery significantly improved lip cant and facial midline deviation in patients with class III asymmetry and lip cant. Mandibular roll correction and pre-treatment lip cant severity were key factors associated with lip cant correction, while chin and mandibular shift correction were associated with facial midline improvement.

## Highlights

• CBCT assessed lip cant and facial midline symmetry.
• Surgery reduced lip cant by 64 % and facial midline deviation by 61 %.
• Mandibular roll correction was associated with lip cant improvement.
• Pre-surgical lip cant severity was strongly associated with correction.
• Chin and mandibular shift corrections improved facial midline symmetry.

## 1 Introduction

Lip cant is a common concern among patients with facial asymmetry [[1], [2], [3]]. The gold standard for correcting lip cant caused by skeletodental asymmetry is orthognathic surgery (OGS) [[3], [4], [5]]. However, the predictability of lip cant reduction following OGS remains limited and challenging.

Three-dimensional (3D) virtual surgical planning has provided quantitative insights into the roles of bimaxillary OGS and maxillary occlusal cant in lip cant correction [6,7], yet findings remain inconsistent [4,[8], [9], [10]]. While posterior occlusal cant correction has been traditionally associated with lip cant improvement [[8], [9], [10]], Kim et al. [4] reported a stronger correlation with anterior occlusal cant changes. Other studies have similarly highlighted the significant role of anterior occlusal cant in lip cant correction [[9], [10], [11]]. Additionally, the response of lip cant correction to maxillary occlusal cant changes varies from 49 % to 66 % [9,12], suggesting that additional factors contribute to successful outcomes.

Conflicting findings have also emerged regarding the correlation between mandibular asymmetry correction and lip cant improvement. While several studies reported a relationship between lip cant correction and menton deviation correction [9,[13], [14], [15]], Suzuki-Okamura et al. [6] found no significant influence of menton deviation or mandibular roll asymmetry on lip cant changes.

These discrepancies underscore the complexity of factors influencing lip cant correction and highlight the need for further research. Advances in 3D imaging with cone-beam CT (CBCT) allow simultaneous evaluation of soft- and hard-tissue structures over time. Therefore, this study aimed to utilize CBCT imaging to assess the impact of bimaxillary surgery on lip cant and facial midline correction, and to identify key factors associated with these improvements in patients with class III asymmetry and lip cant. The research question of the present study, structured using the PICO framework, is as follows: In skeletally mature patients with class III facial asymmetry (Population), does bimaxillary orthognathic surgery (Intervention), compared to their preoperative condition (Comparison), improve soft-tissue symmetry, as reflected by lip cant and facial midline deviation (Outcome)?

## 2 Materials and methods

### 2.1 Study design and sample

This prospective study consecutively recruited adults of skeletal class III deformity with lip cant who underwent bimaxillary surgery from year 2022–2024. Selection criteria were: (1) skeletal class III deformity (ANB <0°) with skeletal menton deviation ≥4 mm and lip cant ≥1 mm; (2) underwent Le Fort I osteotomy and bilateral sagittal split osteotomy (BSSO) setback using a surgery-first approach at the Chang Gung Craniofacial Center by the same team of attending surgeons; (3) the 3D surgical design, and postsurgical orthodontic treatment by a senior orthodontist who treated cases with a surgery-first approach; (4) no genetic syndromes or cleft lip or palate, (5) no history of craniofacial trauma or OGS; and (6) agreed to sign informed consent for the study. The study protocol was approved by the Institutional Review Board of the hospital (202200388B0).

### 2.2 Surgical procedures

All surgeries were performed using a maxilla-first sequence. Le Fort I osteotomy was performed in accordance with the technique described by Chu et al. [16]. The lip or occlusal cant was corrected by either a bilateral asymmetric posterior impaction or a posterior impaction on one side combined with a posterior extrusion on the other side. Bone grafting was routinely performed on the extrusion side [17]. BSSO was performed with the short-split technique modified from Hunsuck [18]. Mandible inferior cortex shaving could be done on the vertically longer side based on surgical simulation image. Osseous genioplasty served as a finishing touch-up to chin symmetry. Except for genioplasty or mandibular contouring, patients received no other surgical intervention. Rigid fixation was performed with mini-plates and monocortical screws. Intermaxillary fixation was removed immediately after surgery. Alar cinch was performed for all patients without any additional soft-tissue treatments.

### 2.3 CBCT images and analysis

CBCT images of all patients were acquired presurgery (T0) and postsurgery follow-up after completion of orthodontic treatment (T1), using an i-CAT 3D Dental Imaging System (Imaging Sciences International, Hatfield, PA). The head was in the natural position. Parameters were: 120 kVp, 0.4 × 0.4 × 0.4-mm voxel size, a 40-s scan time, and a 20 × 20-cm field of view. Prior to the scan, patients were instructed not to swallow, to keep their mouths closed, and to maintain centric occlusion.

Avizo Standard software version 7.1 (FEI, Mérignac, France) rendered data into 3D volumetric images. Sagittal, axial, and coronal slices, as well as 3D reconstructions, were analyzed by a single experienced investigator. Before analysis, 3D images were reoriented as follows: (1) the axial plane, Frankfort horizontal plane (FHP), passed through the right or left (clear side) porion and bilateral orbitale; (2) the mid-sagittal plane (MSP) passed through the nasion, basion, and perpendicular to the FHP; (3) the coronal plane was set perpendicular to the MSP and FHP, passing through the nasion. Subsequently, 3D images were flipped over until all the mentons were deviated to the left side (deviated side); the right side was the opposite side. Cranial structures unaffected by surgery were used to superimpose CBCT images from T0 and T1; image coordinates were aligned with the nasion as the origin. Landmarks on CBCT images [Table 1] were used to measure changes from T0 to T1 regarding facial soft-tissue and dental asymmetry.

**Table 1:** Asymmetry measurements of facial soft tissue and teeth using landmarks assigned on CBCT.

Measurement	Definition
Facial soft tissue	
Lip cant	The absolute difference in vertical distance of bilateral cheilions (Ch)
Nose deviation	The absolute distance between the subnasale (Sn) and mid-sagittal plane (MSP)
Upper lip deviation	The absolute distance between the labial superius (Ls) and MSP
Lower lip deviation	The absolute distance between the labial inferius (Li) and MSP
Chin deviation	The absolute distance between the soft-tissue menton (Me’) and MSP
Facial midline deviation	The sum of the values of nose, upper lip, lower lip, and chin deviations
Teeth	
U1 deviation	The absolute distance between the upper incisor midpoint and MSP
L1 deviation	The absolute distance between the lower incisor midpoint and MSP
Occlusal cant	
Upper anterior	The absolute difference in the vertical distance of bilateral upper canines
Upper posterior	The absolute difference in the vertical distance of bilateral upper first molars
Lower anterior	The absolute difference in the vertical distance of bilateral lower canines
Lower posterior	The absolute difference in the vertical distance of bilateral lower first molars

**Fig. 1:**
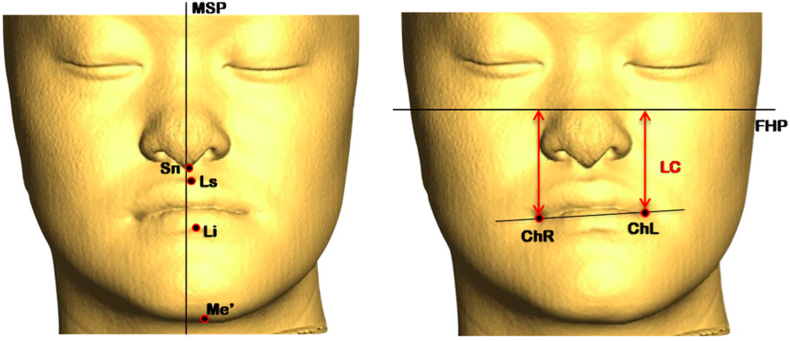
CBCT images with reference planes (MSP, mid-sagittal plane; FHP, Frankfort horizontal plane) and soft-tissue landmarks for measures of midline deviation and lip cant (LC). LC is the absolute difference in vertical distance of bilateral cheilions. Abbreviations: Sn: subnasale; Ls: labial superius; Li: labial inferius; Me:, soft-tissue menton; ChR: right cheilion; ChL: left cheilion.

Changes in symmetry was assessed for lip cant and facial midline deviation (the sum of the values for nose [Sn], upper lip [Ls], lower lip [Li], and chin [Me’] deviations) [Fig. 1]. Changes in incisor deviation were based on the midpoints of the upper and lower central incisors. Changes in upper anterior and posterior occlusal cants were based on the upper canine tips and the mesiobuccal tips of upper first molars, respectively; lower anterior and posterior occlusal cants were based on lower canine tips and mesiobuccal tips of lower first molars.

**Fig. 2:**
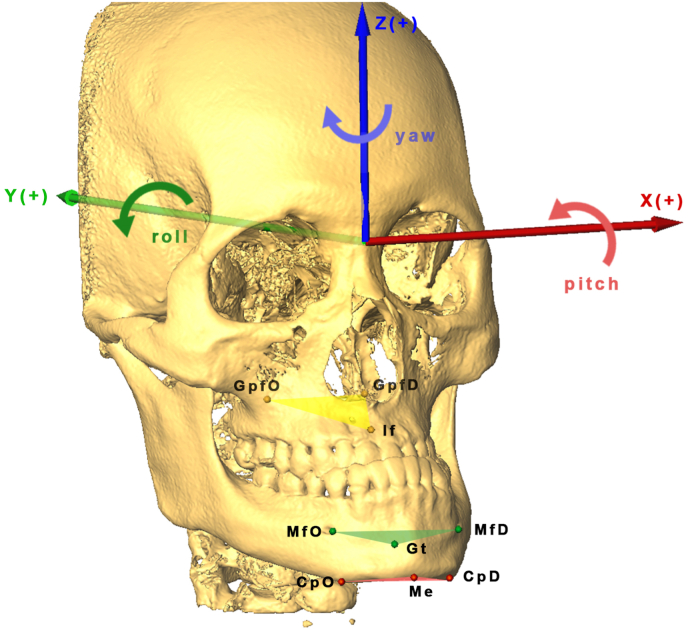
Reconstructed virtual triangles of the maxilla (yellow), mandible (orange), and chin (red). O, side opposite menton deviation; D, side of menton deviation; If, incisive foramen; Gpf, greater palatine foramen; Gt, genial tubercle; Mf, mental foramen; Me, menton; Cp, chin point. Translational movements (arrow): x-axis (left-right), y-axis (posterior-anterior), and z-axis (superior-inferior); + = movement to the left, posterior and superior side. Rotational movements (arrows): x-axis (counter-clockwise pitch, red arrow), y-axis (counter-clockwise roll, green arrow), and z-axis (clockwise yaw, blue arrow). + = counter-clockwise pitch, counter-clockwise roll, and clockwise yaw rotation.

Skeletal changes were evaluated by positional changes in osteotomy segments (maxilla, mandible, and chin) using virtual triangles [17,19,20]. The maxillary triangle included the incisive foramen and bilateral greater palatine foramina. The mandibular triangle included the genial tubercle and bilateral mental foramina. The chin triangle included the menton and bilateral lateral chin points. The translations and rotations of the virtual triangles were calculated through the centroid of each triangle from T0 to T1 [17,19,20] [Fig. 2].

Actual values were used for regression analysis. The midline landmark (Sn, Ls, Li or Me’) on the deviated side and the lip or occlusal cant tilted upward on the deviated side was defined as a positive deviation or cant; the opposite was defined as a negative value.

The intraclass correlation coefficient (ICC), technical error of measurement (TEM) and Dahlberg's formula were calculated to determine intra-examiner reliability. CBCT images from 10 randomly chosen patients were measured twice by the same investigator, with a two-week interval. The mean ICC was 0.970 with a 95 % confidence interval (CI) ranging from 0.964 to 0.975, the mean TEM was 0.172 with a 95 % CI ranging from 0.086 to 0.258, and the mean Dahlberg's formula was 0.185 with a 95 % CI ranging from 0.098 to 0.272, indicating excellent reliability.

### 2.4 Statistical analysis

Power analysis was performed using G∗Power version 3.1.9.7 for Windows (Franz Faul, Universitat Kiel, Germany). To detect significant correction of lip cant after treatment with an alpha level of 0.01 and a power of 0.80, a sample size of 51 subjects is required for within-group comparison.

SPSS version 23.0 for Windows (SPSS Inc, Chicago, USA) was used. Demographic and clinical characteristics are presented as means ± standard deviation (SD) and as frequency (n) and percentage (%). The amount of % correction of lip cant and facial midline deviation was expressed as the mean surgical change at follow-up (T0 – T1) divided by the measure at T0. The Shapiro–Wilk test determined normality of data distribution. Changes in soft-tissue and dental variables from T0 to T1 were examined with paired t–tests. Changes in skeletal position from T0 to T1 were examined with one-sample t-tests. Stepwise linear regression analysis was performed to identify factors associated with the correction of lip cant and facial midline deviation; independent variables included age, gender, initial severity of asymmetry, surgical approach (bilateral asymmetric posterior maxillary impaction vs. a posterior impaction on one side combined with a posterior extrusion on the other side), and surgical changes in dental and skeletal position. To assess potential multicollinearity among independent variables, variance inflation factors (VIFs) were calculated for the final regression models. All VIF values were <2.0, suggesting low collinearity and supporting the stability of the identified factors. Statistical significance was set at *p* < 0.01 to account for multiple comparisons.

A post hoc power analysis was also performed on the association with lip cant correction. With two predictors, a sample size of 50, an observed R^2^ of 0.579, and α = 0.01 (two-tailed), the achieved power was 0.99, indicating adequate power for detecting a statistically significant association.

## 3 Results

Characteristics of the 50 patients are shown in [Table 2]. The mean age was 24.8 ± 5.1 years; 56 % were males. The presurgical mean menton deviation was 7.9 ± 3.6 mm; lip cant was 2.5 ± 1.6 mm. Thirty-four patients received genioplasty for purposes including refining facial profile, adjusting facial height, and improving chin asymmetry. The mean time of postsurgical follow-up was 1.7 ± 0.3 years.

**Table 2:** Demographic and presurgical clinical characteristics of patients (N = 50).

Characteristic	Mean ± SD	n (%)
Gender		
Male		28 (56)
Female		22 (44)
Age at surgery, years	24.8 ± 5.1	
Menton deviation, mm	7.9 ± 3.6	
Menton deviation to the left		29 (58)
SNA,^o^	81.3 ± 4.2	
SNB,^o^	84.7 ± 4.4	
ANB,^o^	−3.5 ± 2.7	
S–N-pogonion,^o^	84.8 ± 3.9	
SN-mandibular plane,^o^	36.8 ± 6.4	
SN-upper incisor axis,^o^	109.7 ± 6.9	
Lower incisor axis-mandibular plane,^o^	80.9 ± 7.8	
Overjet, mm	−1.4 ± 3.1	
Overbite, mm	−0.6 ± 2.7	
Lip cant, mm	2.5 ± 1.6	

SD = standard deviation, S = sella, N = nasion, A = A point, B=B point, Pog = pogonion, SN = sella-nasion line.

Changes in lip cant and facial midline deviation at follow-up are shown in [Table 3]. Significant reductions were seen for lip cant, lower lip deviation, chin deviation and facial midline deviation (1.6 ± 1.6 mm, 2.4 ± 1.7 mm, 5.8 ± 4.2 mm, and 9.7 ± 7.2 mm, respectively). Postsurgical changes in lip cant and facial midline symmetry indicated a correction of approximately 64 % and 61 %, respectively.

**Table 3:** Changes in lip cant and facial midline deviation at postsurgical follow-up (T0 to T1).

Measurement	T0	T1	T0-T1^a^	*P*
	Mean ± SD	Mean ± SD	Mean ± SD	
Lip cant, mm	2.5 ± 1.6	0.9 ± 0.6	1.6 ± 1.6	<0.001
Nose deviation, mm	0.6 ± 0.6	0.9 ± 0.7	−0.2 ± 0.8	0.032
Upper lip deviation, mm	1.4 ± 1.0	1.2 ± 0.9	0.2 ± 1.2	0.239
Lower lip deviation, mm	3.9 ± 1.8	1.5 ± 1.1	2.4 ± 1.7	<0.001
Chin deviation, mm	7.9 ± 3.8	1.9 ± 1.7	5.8 ± 4.2	<0.001
Facial midline deviation, mm	15.9 ± 7.6	6.2 ± 3.5	9.7 ± 7.2	<0.001

T0 = before surgery, T1 = postsurgical follow-up, SD = standard deviation.

a A positive value indicates a postsurgical reduction in the deviation. A negative value indicates a postsurgical increase in the deviation.

[Table 4] presents mean changes in incisor deviation and occlusal cant. Significant improvements were seen for the deviation of lower incisors (3.5 ± 2.3 mm, *p* < 0.001). The cant for the occlusal planes also improved: upper anterior and posterior (0.6 ± 1.3 mm and 0.8 ± 1.5 mm, respectively, *p* < 0.01), and lower anterior and posterior (0.7 ± 1.3 mm and 1.2 ± 1.9 mm, respectively, *p* < 0.001).

**Table 4:** Surgical changes in the positions of teeth.

Measurement	T0	T1	T0-T1^a^	*P*
	Mean ± SD	Mean ± SD	Mean ± SD	
Incisor midpoint				
Upper deviation, mm	1.3 ± 1.1	1.6 ± 1.4	−0.3 ± 1.5	0.199
Lower deviation, mm	4.9 ± 2.2	1.4 ± 1.2	3.5 ± 2.3	<0.001
Occlusal cant				
Upper anterior, mm	1.6 ± 1.3	1.0 ± 0.7	0.6 ± 1.3	0.003
Upper posterior, mm	2.1 ± 1.3	1.4 ± 1.1	0.8 ± 1.5	0.001
Lower anterior, mm	1.6 ± 1.3	0.9 ± 0.8	0.7 ± 1.3	<0.001
Lower posterior, mm	2.5 ± 1.9	1.3 ± 1.0	1.2 ± 1.9	<0.001

T0 = before surgery, T1 = postsurgical follow-up, SD = standard deviation.

a A positive value indicates a postsurgical reduction in the deviation or cant. A negative value indicates a postsurgical increase in the deviation or cant.

Significant changes were observed for most translational or rotational movements of the facial skeleton [Table 5]. Significant translation and rotation occurred in the maxilla, except for left/right shift, and all movements of the mandible were significant. The maxilla primarily moved anteriorly and superiorly, and turned (yaw) and tilted (roll) to the opposite side (all *p* < 0.001). The mandible shifted to the opposite side, moved backward and upward (*p* < 0.001), and turned (yaw) and tilted (roll) to the opposite side (*p*< 0.01 to *P* < 0.001). Chin translational movements and yaw rotation were similar to the mandible (all *p* < 0.001), however there was no significant roll or pitch rotation.

**Table 5:** Surgical changes of skeletal position.

Measurement^a^	Mean ± SD	*P*
Maxilla		
Translation^b^, mm		
Left/Right	−0.5 ± 1.4	0.040
Posterior/Anterior	−2.3 ± 1.9	<0.001
Superior/Inferior	1.7 ± 1.6	<0.001
Rotation^c^, °		
Yaw	1.6 ± 2.7	<0.001
Roll	−1.6 ± 3.4	<0.001
Pitch	−4.4 ± 4.3	<0.001
Mandible		
Translation^b^, mm		
Left/Right	−4.9 ± 3.0	<0.001
Posterior/Anterior	7.0 ± 6.0	<0.001
Superior/Inferior	0.7 ± 2.0	<0.001
Rotation^c^, °		
Yaw	2.2 ± 2.5	<0.001
Roll	−2.5 ± 2.9	<0.001
Pitch	−0.3 ± 6.2	0.002
Chin		
Translation^b^, mm		
Left/Right	−6.8 ± 4.7	<0.001
Posterior/Anterior	6.1 ± 8.0	<0.001
Superior/Inferior	0.5 ± 2.8	<0.001
Rotation^c^, °		
Yaw	2.1 ± 3.3	<0.001
Roll	0.1 ± 4.3	0.013
Pitch	2.2 ± 12.9	0.547

SD = standard deviation.

a The measurements were based on the centroids of triangles of maxilla, mandible, and chin. The maxillary triangle is composed of the incisive foramen and bilateral greater palatine foramina. The mandibular triangle is composed of the genial tubercle and bilateral mental foramina. The chin triangle is composed of the menton and bilateral lateral chin points.

b Translational change: Left/Right: a positive value indicates a postsurgical position more to the left (deviated side); a negative value indicates a postsurgical position more to the right (opposite side). Posterior/Anterior: a positive value indicates a postsurgical position that is more posterior; a negative value indicates a postsurgical position that is more anterior. Superior/Inferior: a positive value indicates a postsurgical position more superior; a negative value indicates a postsurgical position more inferior.

c Rotational change: Yaw: a positive value indicates a postsurgical clockwise rotation around the z-axis; a negative value indicates a postsurgical counter-clockwise rotation around the z-axis. Roll: a positive value indicates a postsurgical counter-clockwise rotation around the y-axis; a negative value indicates a postsurgical clockwise rotation around the y-axis. Pitch: a positive value indicates a postsurgical counter-clockwise rotation around the x-axis; a negative value indicates a postsurgical clockwise rotation around the x-axis.

Stepwise regression indicated changes in roll rotation of the mandible from T0 to T1 and the initial amount of lip cant at T0 were factors associated with significant corrections for lip cant (R^2^ = 0.579, [Table 6]). A second stepwise regression suggested the factors associated with facial midline deviation correction were left/right shift of the chin and mandible from T0 to T1 (R^2^ = 0.845, [Table 6]).

**Table 6:** Stepwise regression analysis: association factors for correction of lip cant and facial midline deviation following bimaxillary surgery.

Model^a^		Unstandardized					
		B	SE	β	T	*P*	R^2^
Lip cant	Constant	1.095	0.258		4.238	<0.001	0.579
	Mandibular roll rotation,^o^ (T0-T1)	0.227	0.069	0.456	3.282	0.002	
	Lip cant, mm (T0)	0.298	0.105	0.394	2.839	0.008	
Facial midline deviation	Constant	1.133	1.039		1.090	0.284	0.845
	Chin translation, left/right, mm (T0-T1)	0.853	0.227	0.495	3.759	0.001	
	Mandibular translation, left/right, mm (T0-T1)	1.221	0.349	0.461	3.502	0.001	

SE = standard error, B = unstandardized beta, β = standardized beta, T0 = before surgery, T1 = postsurgical follow-up.

a Only independent variables with a significance of *p* < 0.01 are included.

## 4 Discussion

Lip or occlusal cant is a common characteristic of class III asymmetry [17,21,22]. Identifying factors associated with lip cant and facial midline deviation correction could enhance surgical planning and improve patient consultations. In the present study, bimaxillary surgery achieved corrections of 64 % in lip cant and 61 % in facial midline deviation. Regression analysis identified mandibular roll correction as a significant factor of lip cant correction. This finding aligns with previous studies showing that mandibular surgery alone [13,14] or bimaxillary surgery [6,8,10,11,15,17] significantly improved lip cant, whereas isolated maxillary surgery did not.

Maxillary occlusal cant correction using Le Fort I roll rotation improves lip cant, a finding well-documented in the bimaxillary surgery literature [4,6,8,11,12]. A study utilizing 2D frontal cephalograms and photographs reported a lip cant correction-to-maxillary occlusal cant correction ratio of 51.5 ± 8.4 % [12], indicating that nearly half of the lip cant correction resulted from surgical correction of maxillary occlusal cant. However, the relationship between lip cant changes and skeletal surgical changes, including translations and rotations, has not been thoroughly investigated. This evaluation is crucial, as maxillary occlusal cant does not always align with maxillary or mandibular cant.

In the present study, Pearson correlation analysis revealed a significant association between changes in maxillary occlusal cant and lip cant (r = 0.67, *p* < 0.001, data not shown). However, regression analysis identified mandibular roll asymmetry correction—not maxillary or mandibular occlusal cant or maxillary roll asymmetry—as the primary factor associated with lip cant reduction. This discrepancy may be attributed to differences in sample size and analyzed variables. While Bergeron et al. [8] and Freudlsperg et al. [11] each analyzed 14 subjects with class III deformity, the current study assessed 50 subjects. Additionally, their studies did not account for maxillary or mandibular translations or rotations. Although Suzuki-Okamura et al. [6] found no significant influence of maxillary or mandibular rotation on lip cant changes, their definition of jaw rotation was two-dimensional (based on the angulation change of bilateral greater palatine foramina or mental foramina) rather than three-dimensional.

Given the apparent influence of mandibular roll correction on lip cant improvement, a mandible-first surgical approach could be considered, particularly in cases where the Le Fort I osteotomy involves a posterior impaction on one side combined with a posterior extrusion on the contralateral side. In such scenarios, repositioning the mandible first might facilitate better alignment of the lower facial structures and subsequently guide maxillary adjustments to optimize facial symmetry. Nevertheless, further studies are needed to validate the benefits of mandible-first sequencing over the conventional maxilla-first protocol. Although mandibular roll correction emerged as a significant factor associated with lip cant improvement, it is important to recognize that lip cant correction is likely a more complex soft-tissue response rather than a direct reflection of skeletal repositioning alone. This notion aligns with prior findings that mandibular symmetry, including menton deviation, is closely associated with lip cant [23]. Soft-tissue adaptation, perioral muscular balance, and scar tissue remodeling may all contribute to the final postoperative outcome [[23], [24], [25]].

Soft tissue adaptation following dentoskeletal changes, particularly concerning lip cant, typically stabilizes within 6–9 months postoperatively. Studies have observed significant changes in lip position and nasolabial angle during this period, with minimal alterations noted between the 6th and 9th months, indicating a plateau in soft tissue adaptation [26,27]. The shortest postsurgical follow-up was 1.1 years; therefore, soft tissue adaptation could be assumed to have been generally completed.

The anatomy of the perioral region provides insight into how mandibular positional changes influence lip cant. Lip cant is defined by the vertical height discrepancy between the bilateral mouth commissures, which is influenced by the vertical proportion of the underlying skeletodental structures and the balance of muscular forces. Upward traction is exerted by the zygomaticus major and levator anguli oris muscles, while downward traction is applied by the depressor anguli oris and depressor labii inferioris muscles [28]. Notably, the maxillary segment is not directly attached to any of these muscles. In contrast, the mandibular distal segment serves as the origin for the depressor anguli oris and depressor labii inferioris. These muscular attachments are located closer to the mental foramen than to the mandibular or maxillary first molar, suggesting that the former may be more relevant in assessing the relationship between skeletal position and lip cant.

In clinical practice, the strategy of overcorrection or undercorrection should be considered according to the underlying etiology of asymmetry. In cleft lip patients, where lip cant partially results from soft tissue deficiency and postsurgical scarring, undercorrection of lip cant is often inevitable despite skeletal realignment. Conversely, in patients with severe lip cant, a mild degree of skeletal overcorrection may be reasonable and beneficial, as suggested by the positive correlation between the amount of correction achieved and initial severity. These considerations should be integrated into surgical planning to optimize both functional and esthetic outcomes. However, the predictive accuracy for the correction of lip cant (R^2^ = 0.579) was lower than that for facial midline deviation (R^2^ = 0.845), confirming the higher ratios of soft-to-hard tissue movements in central facial region compared to lateral facial regions [29]. The stronger prediction observed for facial midline correction likely reflects the more direct relationship between lower lip and soft tissue chin deviation and mandibular body and chin translation, despite asymmetric soft tissue size or shape is another possible contributing factor. Lip cant may also be influenced by additional factors such as soft tissue quantity or tone. Despite significant improvements, approximately 36 % of lip cant and 39 % of facial midline deviation remained after treatment. These residual asymmetries highlight the importance of setting realistic expectations and discussing potential soft-tissue limitations with patients during preoperative consultation.

The lower part of the facial midline, including the transverse position of the lower lip and chin, demonstrated significant postsurgical improvement compared to the midline of the nasal base (subnasale) and upper lip. This suggests that the lower facial structures respond more effectively to jaw correction. Our finding that the correction of chin and mandibular shift asymmetry was associated with improvements in facial midline deviation aligns with previous studies, which reported that transverse changes in the menton influenced the deviation of the subnasale, upper lip, and lower lip following mandibular osteotomy [14,30].

The mentalis muscle intermingles with the lower margin of the orbicularis oris muscle, while the superficial fibers of the orbicularis oris insert into the philtral ridge and Cupid's bow [31]. This anatomical relationship may partly explain the suboptimal correction of subnasale and upper lip deviation observed in the present study, as residual mandibular and chin shift asymmetry could have contributed to these outcomes. Additionally, compromises in nasal and maxillary symmetry are sometimes necessary to achieve optimal mandibular and overall facial symmetry [32].

This study had two notable strengths. First, we utilized 3D-CBCT imaging, which enabled superimposition of pre- and post-surgical images, providing more precise estimations of occlusal cant compared to previous studies that relied on 2D posteroanterior cephalograms and frontal photographs [12,13,33]. Second, the use of 3D-CBCT allowed us, for the first time, to analyze the relationship between postsurgical movements of the maxilla, mandible, and chin and the correction of lip cant and facial midline deviation, considering all six degrees of freedom. Despite these strengths, the study had limitations. It did not directly assess the impact of perioral musculature on lip cant. Additionally, while surgical changes in dental position were evaluated, the dental changes observed at follow-up (mean 1.7 years post-surgery) likely resulted from a combination of surgical and orthodontic treatment. Therefore, these variables reflect the net treatment outcome rather than the isolated effect of surgery—a limitation that might be unavoidable when using a surgery-first approach, unless no orthodontic treatment is initiated within the first 6–9 months after surgery, which would be unethical. However, the dental changes were not related to the lip cant or facial midline deviation in the regression. Future research should also explore facial contour asymmetry to further refine treatment outcomes.

## 5 Conclusion

This study utilized CBCT imaging to evaluate lip cant and facial soft-tissue midline symmetry in 50 patients before and after orthognathic-orthodontic treatment. In patients with class III asymmetry and lip cant, bimaxillary surgery achieved 64 % correction in lip cant and 61 % correction in facial midline deviation. Unlike previous reports, our findings identified mandibular roll asymmetry correction and the pre-treatment severity of lip cant as key factors associated with successful lip cant correction. Additionally, correction of chin and mandibular shift asymmetry emerged as significant factors associated with effective facial midline deviation correction.

## Financial disclosure statement

This research is supported by the 10.13039/100012553Chang Gung Memorial Hospital, Taiwan (CMRPG5M0081-83).

## Declaration of competing interest

The authors have no conflicts of interest relevant to this article.
